# Association between Advanced Glycation End Products and Impaired Fasting Glucose: Results from the SALIA Study

**DOI:** 10.1371/journal.pone.0128293

**Published:** 2015-05-27

**Authors:** Tom Teichert, Anne Hellwig, Annette Peßler, Michael Hellwig, Mohammad Vossoughi, Dorothea Sugiri, Andrea Vierkötter, Thomas Schulte, Juliane Freund, Michael Roden, Barbara Hoffmann, Tamara Schikowski, Christian Luckhaus, Ursula Krämer, Thomas Henle, Christian Herder

**Affiliations:** 1 Institute for Clinical Diabetology, German Diabetes Center, Leibniz Center for Diabetes Research at Heinrich Heine University Düsseldorf, Düsseldorf, Germany; 2 German Center for Diabetes Research (DZD e.V.), Partner Düsseldorf, Düsseldorf, Germany; 3 Institute of Food Chemistry, Technische Universität Dresden, Dresden, Germany; 4 IUF—Leibniz Research Institute for Environmental Medicine at Heinrich Heine University Düsseldorf, Düsseldorf, Germany; 5 Department of Psychiatry and Psychotherapy, Heinrich Heine University Düsseldorf, Düsseldorf, Germany; 6 Department of Endocrinology and Diabetology, Medical Faculty, Heinrich Heine University Düsseldorf, Düsseldorf, Germany; 7 Medical Faculty, Heinrich Heine University Düsseldorf, Düsseldorf, Germany; 8 Swiss Tropical and Public Health Institute, Basel, Switzerland; 9 University of Basel, Basel, Switzerland; CDFD, INDIA

## Abstract

Advanced glycation end products (AGEs) may contribute to the development of type 2 diabetes and related complications, whereas their role in the early deterioration of glycaemia is unknown. While previous studies used antibody-based methods to quantify AGEs, data from tandem mass spectrometry coupled liquid chromatography (LC-MS/MS)-based measurements are limited to patients with known diabetes. Here, we used the LC-MS/MS method to test the hypothesis that plasma AGE levels are higher in individuals with impaired fasting glucose (IFG) than in those with normal fasting glucose (NFG). Secondary aims were to assess correlations of plasma AGEs with quantitative markers of glucose metabolism and biomarkers of subclinical inflammation. This study included on 60 women with NFG or IFG (n = 30 each, mean age 74 years) from the German SALIA cohort. Plasma levels of free metabolites (3-deoxyfructose, 3-deoxypentosone, 3-deoxypentulose), two hydroimidazolones, oxidised adducts (carboxymethyllysine, carboxyethyllysine, methionine sulfoxide) and Nε-fructosyllysine were measured using LC-MS/MS. Plasma concentrations of all tested AGEs did not differ between the NFG and IFG groups (all p>0.05). Associations between plasma levels of AGEs and fasting glucose, insulin and HOMA-IR as a measure of insulin resistance were weak (r between -0.2 and 0.2, all p>0.05). The association between 3-deoxyglucosone-derived hydroimidazolone with several proinflammatory biomarkers disappeared upon adjustment for multiple testing. In conclusion, plasma AGEs assessed by LC-MS/MS were neither increased in IFG nor associated with parameters of glucose metabolism and subclinical inflammation in our study. Thus, these data argue against strong effects of AGEs in the early stages of deterioration of glucose metabolism.

## Introduction

The causes and risk conditions of type 2 diabetes (T2D) are manifold, but on the molecular level they all lead to impaired insulin signalling and/or insulin secretion ultimately resulting in hyperglycaemia. With the loss of glycaemic control, the risk of diabetes comorbidities like macro- and microvascular complications increases. Accumulating evidence supports the relevance of advanced glycation end products (AGEs) in the development and progression of these complications.

Hyperglycaemia, but also hyperlipidaemia and oxidative stress, accelerate formation of AGEs by nonenzymatic glycation reactions [1–3]. Excessive AGE accumulation can deteriorate tissue function by interfering with activities of glycated macromolecules and enzymes and by binding to surface receptors for AGEs (RAGE) on leukocytes triggering oxidative stress and inflammatory responses [4]. Subclinical inflammation in turn represents an important risk factor in the development of T2D and related complications [5–7].

Apart from endogenous AGE formation, smoking tobacco products [8, 9] and consumption of heat-processed food and beverages [10] also increases levels of AGEs and their precursor molecules. It was estimated that the daily intake of AGEs ranges between 25 and 75 mg per day, which are partly absorbed from the gastrointestinal tract [11, 12]. Smoking is related to oxidative stress, upregulation of receptors for advanced glycation end products (RAGE), inflammation and increased AGE formation in extrapulmonary tissue [13].

Methylglyoxal-derived hydroimidazolone (MGH) and Nε-carboxymethyllysine (CML) are likely the most abundant AGEs. Levels of MGH range from 43 to 421 nmol/l in the circulation in healthy and diabetes patients [14], and levels of CML range from 27 to 114 nmol/l. It has to be noted that an absolute quantification of AGEs is challenging, because they depend on both assay type and sample preparation. Due to the complex sample preparation for liquid chromatography assays, epidemiological studies tend to use antibody-based assays to characterise glycated and oxidised protein amounts, which are easy to handle and less expensive. However, these assays often lack specificity.

To date it is not clear to what extent AGEs merely reflect hyperglycaemia or contribute actively to the progression of diabetes. In addition, it is not known whether AGEs contribute only to diabetic complications or are also involved in the early deterioration of glucose homeostasis. Preliminary evidence suggests that AGEs from endogenous glycation reactions or external sources may be involved in beta-cell injury [15] and peripheral insulin resistance [16] and may thereby contribute to the development impaired fasting glucose and impaired glucose tolerance. Of note, that these states of higher diabetes risk are already associated with increased prevalence of micro- and macrovascular diabetes-related complications [17].

To explore the role of AGEs in the context of glucose metabolism and systemic inflammation, this cross-sectional study was established to measure AGE levels by highly sensitive and precise liquid chromatography coupled mass spectrometry (LC-MS)-based methods. This study was designed as pilot study with the primary aim to test the hypothesis that plasma levels of AGEs are already increased in individuals with mild hyperglycaemia (i.e. IFG compared to NFG). As secondary aims, we wanted (i) to assess associations between plasma AGEs and quantitative measures of glucose metabolism (i.e. fasting glucose, fasting insulin levels and HOMA-insulin resistance) and (ii) to investigate correlations between plasma AGEs and plasma levels of biomarkers of subclinical inflammation.

## Study Population and Methods

### Study design and population

We performed a cross-sectional pilot study within a subgroup of 60 non-diabetic and non-smoking women participating in the follow-up examination of the SALIA study in 2008/09 [18]. From 363 women with available plasma samples from this examination, we excluded patients with diabetes because the study focused on mild hyperglycaemia. Current smokers were excluded in order to prevent confounding effects from smoking as major determinant of AGE levels. From the remaining non-smoking women with normal fasting glucose (NFG, n = 178) or impaired fasting glucose (IFG, n = 132) we randomly selected 30 from each group for this study. IFG was defined according to the guidelines of the American Diabetes Association [19]. Written informed consent from all study participants was collected. The study was approved by the ethics board of the Ruhr University in Bochum (Germany).

As described before, anthropometric measurements (height, weight) were conducted according to standardised protocols [20]. Additional data were collected using an extensive interview and questionnaire. Information about the women’s socioeconomic status was assessed by the maximum period of education achieved by the participants or their spouses (<10 years vs. ≥10 years). Classification of smoking habits was performed according to the possible answers “never smokers”, “passive smokers (home, workplace)”, “past smokers” or “current smokers”, with active smokers excluded for this pilot study as described above.

### Measurement of parameters of glucose metabolism

Plasma was stored at -80°C directly after blood collection and used for all assays which have been described in detail before [18]. Fasting plasma glucose levels were determined on a Roche/Hitachi Cobas c 311 analyzer (Basel, Switzerland). Plasma insulin was measured by ELISA (Mercodia, Uppsala, Sweden). As surrogate marker of insulin resistance, the homeostasis model assessment-insulin resistance (HOMA-IR) was calculated as fasting glucose (mmol/L) * fasting insulin (μU/ml) / 22.5.

### Measurement of immune mediators

Plasma levels of high sensitive CRP (hsCRP) were determined on a Roche/Hitachi Cobas c 311 analyzer. Interleukin 6 (IL-6), IL-8, tumour necrosis factor (TNF) α, leptin, IL-1 receptor antagonist (IL-1ra), transforming growth factor β_1_ (TGF-β_1_), total adiponectin, high-molecular-weight (HMW) adiponectin, soluble E-selectin (sE-selectin) and soluble intracellular adhesion molecule 1 (sICAM-1) were measured using Quantikine (IL-1ra, TGF-β_1_, total adiponectin, HMW adiponectin, leptin, sE-selectin, sICAM-1) or Quantikine HS (IL-6, IL-8, TNFα) ELISA kits (R&D Systems, Wiesbaden, Germany) as described [18]. Plasma IL-18 was quantified with the ELISA kit from MBL (Nagoya, Japan). Plasma concentrations of macrophage chemoattractant protein-1 (MCP-1) and interferon gamma-induced protein 10 (IP-10) were assessed by bead-based multiplex assays [18] using a Bio-Plex 200 System controlled by Bio-Rad Bio-Plex Manager Software 6.0 from Bio-Rad Laboratories (Hercules, CA). Bead-based assays (Human Obesity Base Kit and Human Cytokine Custom Premix Kit A) were acquired from R&D Systems.

### Measurement of AGEs

#### Non-protein-bound glucose metabolites by GC-MS

Molecular levels of 3-deoxyfructose (3-DF), 3-deoxypentosone (3-DPs) and 3-deoxypentulose (3-DP) in plasma were measured by gas chromatography coupled with mass spectrometry (GC-MS) as described [21]. First, soluble proteins in plasma were removed by precipitation after adding 10 μL internal standard (IS) of methyl-α-D-galactopyranoside (MGP) and 180 μL acetonitrile/methanol (7:3, v/v) mixture to 100 μL plasma. The solution was stored for 30 minutes on ice and centrifuged for 10 minutes at 4500 g and 4°C. 150 μL of clear supernatant were evaporated overnight to dryness under nitrogen flow at 40°C. The dried mixture was resolved and vortexed with 50 μL hydroxyl amine (10 mg/mL pyridine) agent and again evaporated to dryness under the same conditions as before. The dried compounds were prepared for gas chromatography by adding 50 μL N,O-bis(trimethylsilyl)trifluoroacetamide (BSTFA) and incubating this mixture for 2 hours at room temperature. The samples were diluted to a final volume of 100 μL by adding 50 μL n-hexane. The silylated solution was measured within the next 12 hours.

GC-MS analysis was performed on an Agilent 6890 series gas chromatograph, coupled with a 7683 series injection system as well as a 7683 series autosampler. An Agilent 5973 series mass detector with electron impact ionisation (Palo Alto, CA) was used for mass detection. Details regarding the configuration were described before [21]. The temperature gradient was modified in line with our analytes and temperature increased by 7 K/min.

#### Amino adducts

Plasma proteins were hydrolysed prior mass detection by tandem mass spectrometry coupled liquid chromatography (LC-MS/MS). Enzymatic hydrolysis was performed within 96 hours as described [22]. For this purpose 10 μL IS (^13^C_6_-3-DGH, ^13^C_6_-MGH1, D_2_-CML,^13^C6,^15^N_2_-FruLys), 500 μL 0.02 N HCl and 25 μL pepsine solution were added to 20 μL plasma sample. Oxygen-free atmosphere was realised by perfusing the mixture for 15 seconds and the tube for 10 minutes with nitrogen. After incubating the solution for 24 hours at 37°C, 125 μL potassium-phosphate buffer, 25 μL 0.26 M KOH and 25 μL pronase E solution were added for the second step. The oxygen was removed again from the solution. The mixture was returned to the incubation chamber where it remained for another 24 hours at 37°C. An aliquot of 5 μL prolidase and 4 μL aminopeptidase were added for the last step of hydrolysis. Again oxygen was removed from the reaction tube and was restored at the incubation chamber at 37°C for 48 hours. The hydrolysate was stored at -80°C prior analysis.

The determination of the hydroimidazolones methylglyoxal-derived hydroimidazolone-1 (MGH1), 3-deoxyglucosone-derived hydroimidazolone (3-DGH), oxidised adducts carboxymethyllysine (CML), carboxyethyllysine (CEL), methionine sulfoxide (MetSO) and the precursor molecule Nε-fructosyllysine (FruLys) was performed with the hydrolysed plasma sample. For these measurements an aliquot of 100 μL was taken from the defrosted solution and filtered by a regenerated cellulose (RC) 0.2 μm syringe filter into an HPLC vial with a 200 μL insert. Due to the unstable properties of glycation adducts against atmospheric oxygen, the prepared sample was measured within 12 hours after defrosting.

Parameters for the chromatographic assay and mass spectrometry were adapted from previous studies [23]. We performed measurements on an Agilent 1200 series LC module with an Agilent 6410 series mass detector.

#### Amino acids

To calculate the glycation rate of plasma proteins it was necessary to determine the amino acid levels after enzymatic hydrolysis in the samples. Therefore a 50 μL aliquot was taken from the solution after precipitation and 100 μL of lithium citrate buffer were added. The mixture was stored at 8°C for 12 hours and then centrifuged at 4500 g for 10 minutes to separate the solution from sediments. The clear supernatant was used for analysis on a Sykam S4300 (Sykam, Eresing, Germany) amino acid reaction module for amino acid analysis.

### Statistical analyses

Descriptive statistics were conducted after stratification into participants with NFG and IFG. All continuous variables of inflammation, protein glycation as well as oxidation did not follow a Gaussian distribution and are therefore given as median values and 25^th^/75^th^ percentiles. For further analyses (t-tests, regression and correlation analyses), these variables were log-transformed to the base of 2. Hydroimizalones were determined as molar amount of affected amino acid [nmol/mol]. Further products of protein glycation or oxidation were presented as total amount in plasma [μmol/L].

We applied the parametric Student’s t-test to assess unadjusted differences between both groups (NFG and IFG). Linear regression models were used to assess differences of plasma levels of AGEs or inflammation-related biomarkers between the NFG and IFG groups after adjustment for age, BMI, education, smoking status and passive smoking. Pearson correlation coefficients based on log-transformed variables if appropriate (see above) were computed for associations between AGEs and all other variables (AGEs, immunological mediators and markers for glucose metabolism [fasting glucose, fasting insulin and HOMA-IR]) in plasma. We calculated partial correlations coefficients in addition to adjust for confounding variables.

Post-hoc power was calculated by using G*Power 3.1 in order to evaluate the achieved power of the generated results in this pilot study. Calculations were performed according to the software protocol assuming a two-sided level of significance of α = 0.05 and the achieved effect size between NFG and IFG group for each biomarker.

All provided p values are two-sided. P values <0.05 were considered to indicate statistical significance. All statistical analyses were performed using SAS statistics 9.3 (SAS Institute, Cary, NC).

## Results

### Description of the study population

Characteristics of the study population stratified by fasting glucose levels are summarised in Table 1. Both groups did not differ by age, but women with IFG had a higher body mass index (BMI) than women with NFG (p = 0.0008). As expected, fasting glucose, fasting insulin and HOMA-IR were higher in the IFG group (all p<0.0001). Both groups did not differ with respect to level of education, smoking status and exposure to passive smoking.

**Table 1:** Characteristics of the Study Population Stratified by Fasting Glucose Levels.

Variable	Women with normal fasting glucose	Women with impaired fasting glucose	p
N	30	30	
Age [years]	73.9 ± 2.3	74.7 ± 2.7	0.1916
BMI [kg/m^2^]	26.0 ± 4.0	30.3 ± 4.2	0.0001
Fasting glucose [mg/dl]	90.3 (86.5; 92.5)	110.0 (103.5; 115.5)	<0.0001
Fasting insulin [μU/ml]	5.4 (4.5; 6.7)	7.7 (6.6; 10.3)	<0.0001
HOMA-IR	1.2 (0.9; 1.5)	2.1 (1.9; 2.9)	<0.0001
<10 years education [%]	26.7	34.5	0.7911
Smoking (current/former/never) [%]	0.0/3.3/96.7	0.0/16.7/83.3	0.1945
Passive smoking [%]	26.7	43.3	0.2789

The table shows characteristics of the study population: n = 30 with normal fasting glucose, n = 30 with impaired fasting glucose. Data represent mean values ± standard deviation, median with 25^th^ and 75^th^ percentiles, or percentages. The p value indicates statistical differences between both groups for the respective variables.

### Biomarkers of subclinical inflammation in individuals with NFG and IFG

Plasma concentrations of pro- and anti-inflammatory immune mediators are presented in Table 2. The IFG group showed higher plasma levels of leptin (p<0.0001) and TNFα (p = 0.0292) than the NFG group and a lower ratio of HMW/total adiponectin (p = 0.0085), whereas no significant differences were observed for the other biomarkers. Adjusting for age, BMI, smoking status, passive smoking and education had no effect on these results.

**Table 2:** Plasma Concentrations of Immune Mediators Stratified by Fasting Glucose Levels.

Immune mediator	Women with normal fasting glucose	Women with impaired fasting glucose	p*	p**
Acute-phase protein				
hsCRP [mg/dL]	0.2 (0.1; 0.4)	0.2 (0.2; 0.4)	0.7252	0.6766
Proinflammatory cytokines				
IL-6 [pg/mL]	1.0 (0.8; 2.4)	1.6 (1.0; 2.4)	0.5365	0.5589
IL-18 [pg/mL]	208 (169; 301)	241 (185; 326)	0.2114	0.2172
TNFα [pg/mL]	1.2 (0.9; 1.9)	1.4 (1.1; 2.0)	0.0292	0.0234
Leptin [ng/mL]	12.3 (8.1; 16.3)	21.4 (15.6; 31.7)	<0.0001	<0.0001
Antinflammatory cytokines				
IL-1ra [pg/mL]	218 (165; 274)	256 (182; 322)	0.1770	0.1050
TGF-β1 [ng/mL]	4.8 (3.2; 7.9)	5.6 (3.3; 8.1)	0.9161	0.9365
Total adiponectin [μg/mL]	8.0 (5.9; 11.4)	9.2 (7.4; 12.5)	0.7163	0.8972
HMW adiponectin [μg/mL]	4.9 (3.2; 7.4)	3.9 (2.9; 5.4)	0.1429	0.1092
Ratio HMW/total adiponectin	0.54 (0.48; 0.64)	0.43 (0.34; 0.60)	0.0085	0.0133
Chemokines				
MCP-1/CCL2 [pg/mL]	119 (92; 155)	128 (115; 161)	0.3002	0.2633
IP-10/CXCL10 [pg/mL]	50.2 (38.6; 61.9)	44.6 (35.3; 59.7)	0.8604	0.7448
IL-8/CXCL8 [pg/mL]	10.1 (7.6; 12.6)	11.1 (8.4; 13.1)	0.7134	0.8488
Soluble adhesion molecules				
sE-selectin [ng/mL]	22.6 (20.3; 32.1)	28.2 (23.1; 35.9)	0.5168	0.5684
sICAM-1 [ng/mL]	206 (181; 245)	190 (164; 216)	0.0778	0.0766

Data are given as median with 25th and 75th percentiles;

* unadjusted p value;

** p value adjusted for age, BMI, smoking status, passive smoking and education.

### AGE levels in individuals with NFG and IFG

Plasma concentrations of free metabolites (3-DF, 3-DPs, 3-DP), hydroimidazolones (MGH1, 3-DGH), oxidised adducts (CML, CEL, MetSO) and the precursor molecule FruLys did not differ between the NFG and IFG groups (all p>0.05, Table 3). Adjustment for age, BMI, smoking status, passive smoking and education did not change these results.

**Table 3:** Plasma Concentrations of compounds of protein glycation and oxidation.

Analyte	Women with normal fasting glucose	Women with impaired fasting glucose	p*	p**
Precursor molecule				
FruLys [mM]	3.5 (3.2; 3.8)	3.7 (3.2; 4.0)	0.3866	0.2982
Free metabolites				
3-DF [μM]	2.7 (2.4; 3.7)	2.4 (1.9; 2.9)	0.1836	0.3058
3-DPs [nM]	18.8 (16.0; 21.1)	17.6 (13.5; 20.3)	0.0846	0.1283
3-DP [nM]	705.1 (389.9; 832.3)	720.3 (424.3; 912.9)	0.4756	0.5446
Hydroimidazolones				
MGH1 [nmol/mmol Arg]	178.0 (104.2; 222.6)	200.8 (162.1; 259.3)	0.0763	0.0765
3-DGH [nmol/mmol Arg]	74.8 (28.5; 108.9)	62.5 (28.5; 104.8)	0.9544	0.9012
Oxidated adducts				
CML [nM]	676.6 (554.1; 804.5)	688.0 (599.1; 809.7)	0.4400	0.4505
CEL [nM]	152.8 (137.1; 173.4)	169.0 (135.9; 131.3)	0.1409	0.1669
MetSO [mM]	2.1 (1.7; 3.2)	2.5 (1.7; 2.9)	0.8267	0.9188

Data are given as median with 25th and 75th percentiles;

* unadjusted p value;

** p value adjusted for age, BMI, smoking status, passive smoking and education.

3-DF, 3-deoxyfructose; 3-DPs, 3-deoxypentosone; 3-DP, 3-deoxypentulose; MGH1, methylglyoxal-derived hydroimidazolone 1; 3-DGH, 3-deoxyglucosone-derived hydroimidazolone; CML, carboxymethyl lysine; CEL, carboxyethyl lysine; MetSO, methionine sulfoxide; FruLys, Nε-fructosyllysine.

Table 4 demonstrates that most AGEs were not correlated with each other. We found positive correlations for CML and CEL (r = 0.74), 3-DPs and 3-DF (r = 0.48), MGH1 and CML (r = 0.38) as well as MetSO and FruLys (r = 0.29), whereas a negative correlation was observed for FruLys and MGH1 (r = -0.32).

**Table 4:** Correlation Between Plasma Levels of AGEs.

		3-DPs	3-DP	MG H	3-DG H	CML	CEL	MetSO	FruLys
**3-DF**	r	0.4791	-0.2235	-0.2551	-0.0773	-0.1212	-0.1123	-0.0766	-0.0766
	p	0.0001**	0.0917*	0.0533*	0.5642	0.3647	0.4013	0.5676	0.5676
	n	58	58	49	37	58	57	58	58
**3-DPs**	r		-0.0879	-0.1911	-0.1295	0.0697	0.1286	0.1296	0.0026
	p		0.5115	0.1508	0.3328	0.6033	0.3362	0.3323	0.9846
	n		58	49	37	58	57	58	58
**3-DP**	r			0.1027	0.2168	0.1444	0.2084	0.0865	0.0945
	p			0.4430	0.1021	0.2795	0.1164	0.5183	0.4805
	n			49	37	58	57	58	58
**MG H**	r				0.0035	0.3754	0.0765	-0.1113	-0.3191
	p				0.9786	0.0031**	0.5615	0.3970	0.0130**
	n				34	51	50	51	51
**3-DG H**	r					0.1871	0.0981	-0.0899	-0.2521
	p					0.1523	0.4561	0.4946	0.0520*
	n					38	38	38	38
**CML**	r						0.7397	-0.1021	0.0160
	p						<.0001**	0.4374	0.9034
	n						59	60	60
**CEL**	r							0.1856	0.1672
	p							0.1557	0.2017
	n							59	59
**MetSO**	r								0.2907
	p								0.0243**
	n								60

Correlation coefficients r are presented with their corresponding p value. n, number of observations;

** p value <0.05;

* p-value between 0.05 and 0.1.

### Association of plasma AGEs with parameters of glucose metabolism and subclinical inflammation

Associations between plasma levels of AGEs and fasting glucose, fasting insulin and HOMA-IR were weak (r between -0.2 and 0.2) and not statistically significant (all p>0.05) (Table 5). We found some evidence that particularly 3-DGH may be positively correlated with several proinflammatory biomarkers, but adjustment for multiple tests (12x18 correlations in Table 5) rendered these associations not significant anymore.

**Table 5:** Correlation Between AGEs, Glucose, Insulin and Biomarkers of Subclinical Inflammation.

		Fastingglucose	Fasting insulin	HOMA-IR	hsCRP	IL-6	IL-18	TNFα	Leptin	IL-1ra	TGF -β1	Total adiponectin	HMW adiponectin	HMW/total adiponectin	MCP-1	IP-10	IL-8	sE-Selectin	sICAM-1
**Fasting**	r	-	**0.603**	**0.761**	-0.047	-0.012	0.223	-0.016	0.187	-0.004	0.117	-0.256	**-0.315**	-0.157	0.216	-0.050	0.218	0.060	-0.236
**glucose**	p	-	**<.0001**	**<.0001**	0.738	0.933	0.105	0.909	0.177	0.979	0.402	0.062	**0.020**	0.258	0.131	0.729	0.114	0.664	0.085
	n	-	60	60	60	60	60	60	60	60	60	60	60	60	60	60	60	60	60
**Fasting**	r	**0.603**	-	**0.977**	-0.014	-0.079	**0.360**	-0.187	0.248	0.169	0.257	**-0.390**	**-0.452**	-0.197	0.246	-0.042	0.176	0.149	-0.124
**insulin**	p	**<.0001**	-	**<.0001**	0.922	0.575	**0.008**	0.177	0.070	0.221	0.061	**0.004**	**0.001**	0.152	0.084	0.774	0.203	0.282	0.370
	n	60	-	60	60	60	60	60	60	60	60	60	60	60	60	60	60	60	60
**HOMA-IR**	r	**0.761**	**0.977**	-	-0.024	-0.067	**0.353**	-0.156	0.252	0.137	0.240	**-0.387**	**-0.453**	-0.203	0.257	-0.047	0.202	0.138	-0.165
	p	**<.0001**	**<.0001**	-	0.865	0.632	**0.009**	0.260	0.066	0.324	0.080	**0.004**	**0.001**	0.141	0.072	0.746	0.143	0.321	0.233
	n	60	60	-	60	60	60	60	60	60	60	60	60	60	60	60	60	60	60
**3-DF**	r	0.066	-0.032	-0.009	0.189	0.031	-0.051	-0.048	0.085	-0.047	**-0.391**	-0.038	0.065	0.142	-0.040	-0.022	-0.204	-0.135	-0.099
	p	0.643	0.820	0.950	0.181	0.828	0.719	0.734	0.550	0.740	**0.004**	0.787	0.648	0.317	0.787	0.885	0.146	0.341	0.484
	n	58	58	58	58	57	58	58	58	58	**58**	58	58	58	54	54	58	58	58
**3-DPs**	r	-0.015	0.157	0.124	-0.052	-0.121	-0.231	-0.175	0.193	0.212	-0.140	-0.165	-0.157	-0.034	-0.039	-0.045	-0.106	-0.024	-0.007
	p	0.914	0.266	0.381	0.715	0.399	0.100	0.214	0.171	0.132	0.324	0.243	0.266	0.813	0.791	0.763	0.455	0.869	0.960
	n	58	58	58	58	57	58	58	58	58	58	58	58	58	54	54	58	58	58
**3-DP**	r	0.000	0.080	0.065	-0.051	-0.060	-0.088	0.105	-0.089	0.091	0.047	-0.116	-0.187	-0.136	0.000	0.120	0.100	0.068	0.013
	p	0.999	0.573	0.647	0.719	0.673	0.536	0.459	0.532	0.522	0.740	0.414	0.185	0.336	0.998	0.417	0.481	0.632	0.928
	n	58	58	58	58	57	58	58	58	58	58	58	58	58	54	54	58	58	58
**MG H**	r	-0.044	0.207	0.157	-0.072	0.004	0.093	0.080	0.054	0.033	0.146	0.073	-0.011	-0.103	0.073	-0.014	-0.148	0.208	-0.016
	p	0.753	0.133	0.258	0.606	0.980	0.503	0.564	0.699	0.813	0.292	0.601	0.939	0.461	0.614	0.921	0.284	0.132	0.906
	n	51	51	51	51	50	51	51	51	51	51	51	51	51	47	47	51	51	51
**3-DG H**	r	-0.037	0.070	0.047	0.264	**0.271**	**0.276**	-0.152	**0.302**	0.207	0.074	-0.012	0.042	0.076	0.099	0.225	0.133	0.227	0.112
	p	0.788	0.617	0.738	0.053	**0.049**	**0.044**	0.271	**0.026**	0.133	0.594	0.933	0.761	0.584	0.496	0.117	0.337	0.099	0.420
	n	38	38	38	38	**38**	**38**	38	**38**	38	38	38	38	38	35	35	38	38	38
**CML**	r	-0.088	0.035	0.005	0.087	0.062	-0.069	0.071	-0.084	0.059	0.127	-0.094	-0.001	0.111	-0.096	-0.019	-0.151	0.176	-0.181
	p	0.528	0.804	0.974	0.534	0.661	0.620	0.612	0.544	0.671	0.361	0.499	0.997	0.423	0.507	0.897	0.276	0.202	0.191
	n	60	60	60	60	59	60	60	60	60	60	60	60	60	56	56	60	60	60
**CEL**	r	-0.013	0.081	0.062	0.036	-0.150	-0.143	-0.009	-0.033	-0.050	0.074	-0.044	0.044	0.117	-0.193	-0.089	-0.218	-0.058	**-0.407**
	p	0.925	0.560	0.654	0.795	0.284	0.302	0.948	0.811	0.720	0.596	0.751	0.752	0.398	0.180	0.541	0.113	0.677	**0.002**
	n	59	59	59	59	58	59	59	59	59	59	59	59	59	55	55	59	59	**59**
**MetSO**	r	0.076	0.012	0.030	**0.282**	-0.015	-0.042	-0.002	0.055	-0.006	-0.239	0.120	0.068	-0.043	0.069	0.132	-0.155	-0.210	-0.089
	p	0.587	0.933	0.830	**0.039**	0.913	0.762	0.990	0.695	0.964	0.082	0.389	0.625	0.758	0.635	0.359	0.264	0.128	0.523
	n	60	60	60	**60**	59	60	60	60	60	60	60	60	60	56	56	60	60	60
**FruLys**	r	-0.125	-0.172	-0.174	0.033	-0.179	-0.112	0.035	-0.147	0.036	-0.268	0.070	0.156	0.145	-0.184	0.008	0.020	**-0.279**	-0.014
	p	0.366	0.214	0.209	0.812	0.199	0.421	0.802	0.290	0.826	0.050	0.613	0.259	0.294	0.200	0.957	0.885	**0.041**	0.918
	n	60	60	60	60	59	60	60	60	60	60	60	60	60	56	56	60	**60**	60

n, number of observations; p, p-value adjusted for age, BMI, smoking status, passive smoking and education; r, correlation coefficient. Associations with p<0.05 are shown in bold print.

## Discussion

This pilot study analysed the associations between AGEs and mild hyperglycaemia in study participants with NFG and IFG. Plasma AGE levels of FruLys, 3-DF, 3-DPs, 3-DP, MGH1, 3-DGH, CML, CEL and MetSO were independent of fasting glycaemia as well as fasting insulin and HOMA-IR as a surrogate of insulin resistance. Furthermore, correlations between plasma AGE concentrations and biomarkers of subclinical inflammation were rather weak in unadjusted analyses and after adjusting for age, BMI, passive smoking, history smoking and social status.

### LC-MS/MS based measurements of AGEs in plasma

Protein-bound AGEs were detected by LC-MS/MS based measurements, which are characterised by their high specificity and sensitivity [24, 25]. Antibody-based AGE detection was established in proteomic studies because of their easy and less time consuming sample preparation. Reproducible results were obtained not only for CML, but also for levels of pyrraline [26] and methylgloyxal-modified proteins [27–30] by highly specified immunochemical methods. A brief overview of commonly used antibodies for AGE determination is provided by Nagai and Horiuchi [31]. However, the accuracy of these immunoassays depends on the matrix [24, 25, 32] and the specificity of the antibodies. Several studies revealed that most of the antibodies used for AGE measurements show some degree of cross-reactivity with other epitopes [25, 33] and may thus yield misleading results.

Compared to previous works we observed overall lower AGE levels [23] as well as lower concentrations for α-dicarbonyl compounds, but higher levels for 3-DF in plasma compared to previous studies [34, 35]. This discrepancy may have resulted from differences between the evaluated populations and from differences in preanalytical aspects of sample treatment [36].

With respect to free glucose metabolites, we demonstrate the presence of 3-DP in human plasma. This result indicates that ribose or ribose-5-phosphate (R5P) may undergo *in vivo* Maillard reaction as well [37] or that *in vivo* glucosone decomposition results in 3-DPs [38]. The susceptibility of R5P to undergo Maillard reaction might be several times increased compared to glucose-6-phosphate (G6P), as the percentage of open chain carbohydrates is many times higher at R5P than at G6P [39, 40]. Despite the reactivity of R5P, the concentrations of 3-DPs may be lower than levels of 3-DG, because the physiological R5P concentration reaches only up to 10% of the physiological G6P concentration.

### Associations between AGEs and parameters of glucose metabolism

We observed no statistically significant differences in plasma AGEs levels between the NFG and IFG groups. However, we observed a trend towards higher MGH1 levels in IFG.

Our finding is in line with results from previous studies in healthy young adults, elderly people and even T2D patients [41–43]. One of the aforementioned studies determined circulating levels of CEL, CML and the cross-linked pentosidine [42]. However, this study found no association between AGEs when analysed separately or combined and markers of glucose metabolism. But there are also conflicting results, suggesting a possible relationship between AGEs, insulin resistance and insulin secretion. Several research groups examined children and healthy adults [44–47] as well as diabetes patients without and with comorbidities [48–50] and reported positive relationships between by ELISA-measured AGEs and markers of glucose metabolism. The discrepancy between these results could origin from nutrition, as AGE plasma levels will increase after dietary intake [16, 51–53] and thus relationships between circulating AGEs with glucose homeostasis could emerge [50]. Unfortunately, detailed data for dietary habits were not available in the SALIA cohort.

The trend towards higher MGH1 levels in the IFG group is interesting, because previous studies found increased MGH1 levels in patients with type 2 diabetes [28] and type 1 diabetes [54]. These studies indicate that MGH1 levels and impaired glucose metabolism are indeed related, but it is currently unclear to what extent this link is mediated by fasting or postprandial hyperglycaemia and which role the duration of impaired glucose metabolism plays in this context.

We performed a post-hoc power calculation which revealed a power between 5 and 39% to detect significant differences between the NFG and IFG groups for the tested AGEs. Assuming that the mean values and standard deviations we measured reflect data in larger populations, study samples between 200 and 800 individuals would be required to reveal statistical group differences at α = 0.05 and β = 0.8 e.g. for the free metabolites 3-DF, 3-DPs and 3-DP. This information should be helpful for the planning of subsequent studies assessing the relationship between AGEs and hyperglycaemia.

### Associations between AGEs and biomarkers of subclinical inflammation

We also evaluated the associations between plasma AGEs and circulating biomarkers for subclinical inflammation. Women with NFG and IFG showed some differences with respect to levels of inflammation-related biomarkers (leptin, TNFα, ratio of HMW/total adiponectin) as expected. Differences in other diabetes-related cytokines as IL-6 and IL-18 were less pronounced and did not reach statistical significance, which may reflect the moderate sample size of this pilot study. When assessing associations with AGEs we considered several important confounders in our extended adjusted models like age, BMI, smoking status (passive or former) and social status. We observed positive, but rather weak correlations between 3-DGH and proinflammatory IL-6, IL-18 as well as leptin. This finding is supported by previous studies in man, reporting no association between CML and pro- and anti-inflammatory mediators as well as molecules for endothelial cell activation [41, 43, 49, 55]. However, an upregulating effect of AGEs on inflammatory biomarkers was described for dietary ingested AGEs [45, 55]. Furthermore, findings on AGE effects are conflicting as other studies reported significant positive associations between CML and CRP as well as IL-6 in diabetic participants [44] and even negative associations between CML and IL-6 in schoolchildren [47]. However, available data about associations between AGEs, especially for single compounds from this large group, are scarce and inhomogeneous.

In addition, MetSO which can be considered as marker of oxidative stress was associated with increased plasma hsCRP levels [56]. We observed inverse and weak correlations between TGF-β_1_ and 3-DF as well as MetSO and FruLys, but not 3-DGH. TGF-β_1_ may be able reduce reactive oxygen species [57] and thus oxidative stress, but TGF-β_1_ also possesses a fibrotic potential which was associated with the presence of AGEs in plasma and kidney matrix proteins [58–60]. Furthermore, we observed a rather weak and negative association between lysine modifications and the cell adhesion molecule sICAM-1. Both structures are thought to be involved in foam cell formation and atherosclerotic lesions [61, 62], but a possible mechanism linking AGEs and sICAM-1 levels has not yet described.

### Strengths and limitations

Our study has several strengths and limitations. First, this study extends the currently available literature regarding the association between AGEs, their precursors and inflammatory biomarkers in the circulation. To our knowledge, this is the first study assessing the relationship between plasma AGEs and subclinical inflammation in nondiabetic study participants. Second, we measured a variety of immunological mediators in plasma, which are known indicators of systemic subclinical inflammation reflecting mainly cardiometabolic risk. Third, we used precise LC-MS/MS measurements instead of antibody-based assays and thus we were able to measure exact concentrations of each analyte. Furthermore we adjusted our statistical analyses for potential confounders.

There are also several potential limitations that should be taken into account when interpreting our results. We performed a cross-sectional pilot study. Fasting glucose levels have not been measured in previous SALIA examinations so that we could not analyse associations between AGE levels and duration of IFG. Furthermore, we examined elderly women. This could attenuate certain findings due to a survivor effect (individuals with high sensitivity to air pollution and high levels of inflammation may have died earlier than less susceptible women). Data on renal function were not available so that the potential relevance of renal clearance on circulating AGE levels could not be assessed. We also had no information on dietary habits of the study participants. Finally, our study sample was small, so that we may have missed associations due to limited power (type II error). However, our measurements enabled us to estimate the sample sizes needed to detect differences between the NFG and IFG groups based on our observations and may therefore be helpful in the planning of future studies in this field.

### Conclusion

After adjusting for potential confounders, circulating plasma protein glycation and oxidation products as well as glycation intermediates were not increased among elderly women with IFG compared to women with NFG. AGEs were also not associated with markers of subclinical inflammation in this study sample of elderly women. Our post-hoc power calculation indicates that larger studies are required to reveal associations between AGEs measured using LC-MS/MS-based methods and early deterioration of glycaemic control before the onset of T2D.
